# Strong Electron‐Phonon Coupling Mediates Carrier Transport in BiFeO_3_

**DOI:** 10.1002/advs.202301057

**Published:** 2023-05-23

**Authors:** Zhenwei Ou, Bin Peng, Weibin Chu, Zhe Li, Cheng Wang, Yan Zeng, Hongyi Chen, Qiuyu Wang, Guohua Dong, Yongyi Wu, Ruibin Qiu, Li Ma, Lili Zhang, Xiaoze Liu, Tao Li, Ting Yu, Zhongqiang Hu, Ti Wang, Ming Liu, Hongxing Xu

**Affiliations:** ^1^ School of Physics and Technology Center for Nanoscience and Nanotechnology and Key Laboratory of Artificial Micro‐ and Nano‐structures of Ministry of Education Wuhan University Wuhan 430072 China; ^2^ Electronic Materials Research Laboratory Key Laboratory of the Ministry of Education & International Center for Dielectric Research School of Electronic Science and Engineering Xi'an Jiaotong University Xi'an 710049 China; ^3^ Key Laboratory of Computational Physical Sciences (Ministry of Education) Institute of Computational Physical Sciences Fudan University Shanghai 200433 China; ^4^ Key Laboratory of Material Physics Ministry of Education School of Physics and Microelectronics Zhengzhou University Zhengzhou 450001 China; ^5^ Center for Spintronics and Quantum Systems State Key Laboratory for Mechanical Behavior of Materials Department of Materials Science and Engineering Xi'an Jiaotong University Xi'an 710049 China; ^6^ Wuhan Institute of Quantum Technology Wuhan 430206 China; ^7^ School of Microelectronics Wuhan University Wuhan 430072 China

**Keywords:** BiFeO3, carrier transport, electron‐phonon coupling, transient absorption microscopy

## Abstract

The electron‐phonon interaction is known as one of the major mechanisms determining electrical and thermal properties. In particular, it alters the carrier transport behaviors and sets fundamental limits to carrier mobility. Establishing how electrons interact with phonons and the resulting impact on the carrier transport property is significant for the development of high‐efficiency electronic devices. Here, carrier transport behavior mediated by the electron‐phonon coupling in BiFeO_3_ epitaxial thin films is directly observed. Acoustic phonons are generated by the inverse piezoelectric effect and coupled with photocarriers. Via the electron‐phonon coupling, doughnut shape carrier distribution has been observed due to the coupling between hot carriers and phonons. The hot carrier quasi‐ballistic transport length can reach 340 nm within 1 ps. The results suggest an effective approach to investigating the effects of electron‐phonon interactions with temporal and spatial resolutions, which is of great importance for designing and improving electronic devices.

## Graphical abstract

The hot carriers generated in BiFeO_3_ are capable of coupling with phonons and undergo quasi‐ballistic transport over a distance of 340 nm in the first picosecond. Even after undergoing cooling processes, the carrier diffusion coefficient mediated by electron‐phonon coupling remains significantly higher than that of bulk. These findings introduce novel perspectives for the development of efficient electronic devices based on BiFeO_3_.

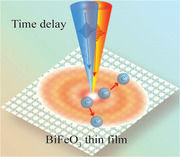

## 1 Introduction

The electron‐phonon interaction has been of great interest in solid‐state physics, notably as one of the major mechanisms determining the electrical resistance,^[^ ^1^ ^]^ carrier mobility,^[^ ^2^, ^3^ ^]^ and high‐temperature superconductivity^[^ ^4^, ^5^ ^]^ in metals and semiconductors. Among these physical properties, the carrier transport and decay characteristics have received much attention as they are crucial for the performance of electronic and optoelectronic devices.^[^ ^6^, ^7^, ^8^ ^]^ The electron‐phonon interactions can alter the carrier transport and set fundamental intrinsic limits to carrier mobilities through various mechanisms.^[^ ^9^ ^]^ For instance, the electrons can be scattered by phonons, leading to temperature (*T*) ‐dependent carrier mobility (*μ*) of *μ* ≈ *T^m^*, where the coefficient *m* varies under different temperatures and electron densities.^[^ ^10^ ^]^ In organic semiconductors, excitons are localized and polarons are formed due to the strong interactions between electrons and vibrational modes, which can then lead to the modulation of carrier mobility and coherent transport range.^[^ ^11^ ^]^ These works indicate that carrier transport property can be mediated by electron‐phonon interactions, and significant improvements in carrier mobility are anticipated with well‐designed electron‐phonon coupling.

Given its importance, electron‐phonon coupling has been extensively studied in a variety of materials.^[^ ^4^, ^12^, ^13^, ^14^ ^]^ Among them, the multiferroics exhibit strong charges‐spins‐lattices interaction and inherent couplings between simultaneous ferroelectric and ferromagnetic orderings.^[^ ^15^, ^16^, ^17^ ^]^ These couplings can be readily manipulated by optical excitations, making multiferroics an outstanding platform for the investigation of electron‐phonon coupling. The bismuth ferrite (BiFeO_3_), the most widely studied room‐temperature multiferroic,^[^ ^18^, ^19^ ^]^ exhibits unique physical properties arising from the inherent couplings and exchange interactions such as magnetoelectric coupling,^[^ ^20^, ^21^ ^]^ photoinduced mechanical strain,^[^ ^22^, ^23^, ^24^ ^]^ and magnon sidebands.^[^ ^25^, ^26^ ^]^ Moreover, unlike most multiferroics, the BiFeO_3_ exhibits strong photoresponse,^[^ ^27^, ^28^ ^]^ photovoltaic effect,^[^ ^28^ ^]^ and domain wall conductivity,^[^ ^29^ ^]^ making it a potential material for optoelectronic and photovoltaic device applications.^[^ ^30^ ^]^ In addition, the carrier decay dynamics can also be modulated by coupling to the spins and optically excited phonons.^[^ ^31^, ^32^ ^]^ Despite the outstanding photoresponse and exchange interactions in BiFeO_3_, the direct impact of electron‐phonon coupling on the spatial and temporal charge carrier transport properties is still a subject to be investigated currently. Understanding how and to what extent electron‐phonon coupling can alter the carrier transport behavior in space and time is important for the development of high‐efficiency electronic devices and may even enable new ways to manipulate electrons and phonons for novel device applications.

**Figure 1:**
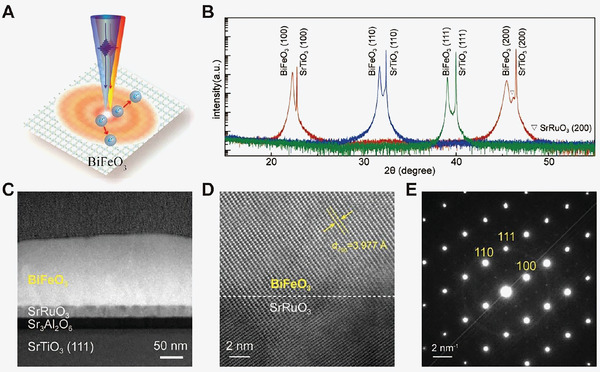
Structural characterization of BiFeO_3_ thin films. A) Schematic representation of the pump‐probe experiments and the carrier transport in the BiFeO_3_ thin film. The incident pump and probe beams are normal to the surface. B) X‐ray diffraction patterns of as‐grown BiFeO_3_ epitaxial thin films. C) Cross‐sectional TEM image of BiFeO_3_ (111) epitaxial thin films. D) HAADF‐STEM image of the SrRuO_3_/BiFeO_3_ interface. E) SAED pattern of BiFeO_3_ (111) epitaxial thin film. It shows the thin film is (111)‐oriented.

To address this issue, transient absorption microscopy (TAM), a microscopy technique combined with ultrafast optical spectroscopy, has been employed to directly study the carrier decay dynamics and image the carrier population distribution in space and time (**Figure** **1**A).^[^ ^33^ ^]^ Here, it illustrates that the carrier transport behavior can be strongly mediated by the electron‐phonon coupling in BiFeO_3_. With photoexcitation, the GHz longitudinal acoustic (LA) phonons are generated by the inverse piezoelectric effect. This LA phonon mode can couple with the photoexcited carriers, resulting in oscillatory properties in carrier decay dynamics and spatial density distribution. Interestingly, quasi‐ballistic transport behavior and doughnut shape carrier distribution are observed within 1 ps, which are caused by the coupling between the hot carrier and phonon mode. It shows that the quasi‐ballistic transport length of ≈340 nm in BiFeO_3_ thin films is significantly larger than that of hot carriers in Si, GaN, and CH_3_NH_3_PbI_3_ perovskites.^[^ ^34^, ^35^, ^36^ ^]^ After the cooling of hot carriers, the maximum diffusion coefficient mediated by electron‐phonon coupling can still be two orders of magnitude larger than that in bulk BiFeO_3_. The long‐range carrier quasi‐ballistic transport over hundreds of nanometers associated with unique multiferroic and photovoltaic properties make BiFeO_3_ potential material for novel device applications combing the ferroelectric, electronic, and optoelectronic functionalities.

## 2 Results

### 2.1 Thin‐Film Growth and Characterization

BiFeO_3_ epitaxial thin films were fabricated by pulsed laser deposition and further details on the sample growth can be found in the Method section. For a systematic study, three epitaxial thin films have been fabricated with different orientations. Figure 1B shows the X‐ray diffraction (XRD) patterns for the three BiFeO_3_ epitaxial thin films on different SrTiO_3_ substrates ((100), (110), and (111) orientations). To improve the epitaxial crystalline quality, a Sr_3_Al_2_O_6_ buffer layer has been introduced before BiFeO_3_ deposition.^[^ ^37^, ^38^, ^39^ ^]^ It is found that all the epitaxial BiFeO_3_ thin films exhibit excellent epitaxy relation on SrTiO_3_. Correspondingly, only (100)/(200), (110), and (111) diffraction peaks can be observed in the detected 2*θ* range, respectively. Thus, it is confirmed that high‐quality epitaxial thin films have been deposited with three different orientations. To further verify the quality of the epitaxial thin films, atomic force microscope (AFM), reciprocal space mapping, and transmission electron microscopy (TEM) measurements have been conducted. Figure S1C (Supporting Information) illustrates the AFM image of the (111)‐oriented BiFeO_3_ epitaxial thin film. It presents a dense microstructure and relatively smooth surface without ordinary defects such as pin holes. The average roughness (*R_a_*) and the square root roughness (*R_q_*) are ≈1.11 and 1.41 nm, respectively. The sharp peaks of the rocking curves present the single‐crystalline properties of the BiFeO_3_ thin films (Figure S3, Supporting Information). The diffraction peaks of the BiFeO_3_ layers follow that of the SrTiO_3_ layers in both conventional X‐ray diffractions (Figure 1; Figure S3, Supporting Information) and reciprocal space mappings (Figure S3, Supporting Information), indicating excellent epitaxial relation. Figure 1C shows the cross‐sectional TEM images of BiFeO_3_ (111) epitaxial thin film. It has distinctly illustrated the SrTiO_3_ substrate, Sr_3_Al_2_O_6_ buffer layer, SrRuO_3_ electrode, and the BiFeO_3_ thin film. The film thickness of the Sr_3_Al_2_O_6_ buffer layer is ≈23 nm and it is ≈142 nm for the BiFeO_3_ (111) epitaxial layer. In addition, Figure 1D shows the magnified high‐angle annular dark‐field (HAADF) image of the SrRuO_3_/BiFeO_3_ interface. It demonstrates a sharp interface and the perfect epitaxy structure between them. The selected area electron diffraction (SAED) pattern of the BiFeO_3_ thin film is shown in Figure 1E which further indicates the orientation is along the (111) plane and the epitaxial relation. Moreover, the lattice constant of BiFeO_3_ can be estimated from the SAED pattern as *a* = 3.946 Å and *β*  = 89.6° with a typical R‐phase.^[^ ^40^, ^41^ ^]^ Figure S2 (Supporting Information) shows the piezo response force microscopy images of the three thin films, which illustrate the ferroelectric properties and domain structures.

The steady‐state absorption and photoluminescence (PL) spectra of (111) BiFeO_3_ thin film are shown in the Supporting Information (Figure S4, Supporting Information). For better understanding, the band structure is marked out by the blue and red‐shaded areas according to results from previous works.^[^ ^25^, ^26^, ^42^ ^]^ The spectra show a sharp increase in absorbance and a strong peak in PL at ≈2.66 eV. This indicates that the bandgap is ≈2.66 eV, which is consistent with previous experimental results.^[^ ^25^, ^42^, ^43^ ^]^ However, the absorptance onset occurs at a much lower energy of ≈1.65 eV. In addition, the absorptance spectrum exhibits a broad shoulder centered at ≈2.0 eV and a small absorbance feature below 1.5 eV. These absorption bands can be assigned to the ^6^ *A*_1*g*_ → ^4^ *T*_1*g*_ (below 1.5 eV) and ^6^ *A*_1*g*_ → ^4^ *T*_2*g*_ (between 1.65 and 2.2 eV) transitions of the Fe^3+^ ions.^[^ ^25^, ^26^ ^]^ These broad absorption bands are named magnon sidebands.^[^ ^25^, ^26^ ^]^ The magnon sidebands originate from the spin‐charge coupling interactions and are associated with the reduced symmetry in BiFeO_3_.^[^ ^25^ ^]^ The small PL peak at ≈1.65 eV can be attributed to the carrier recombination in magnon sidebands. Therefore, it provides an excellent platform to study the spin‐charge‐lattice interactions in multiferroics.

### 2.2 Ultrafast Carrier Dynamics and Electron‐Phonon Coupling

**Figure 2:**
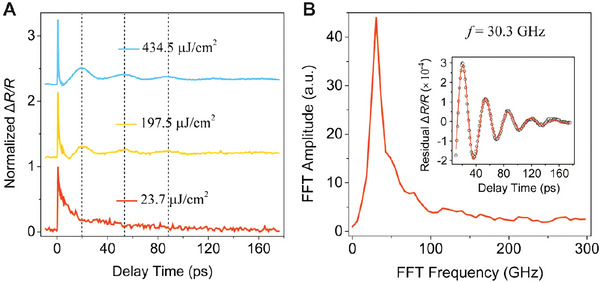
Transient reflection dynamics of BiFeO_3_ thin film. A) Transient reflection spectra under different pump fluence. The pump and probe wavelengths are 400 and 750 nm, respectively. The signals show clear oscillatory characteristics as marked by dashed lines. B) The oscillatory component extracted from the transient reflection signal (inset) and its fast Fourier transform spectrum.

With the broad absorption spectrum, the hot carrier cooling and carrier dynamics can be systematically studied by pump‐probe spectroscopy. In the transient absorption experiments, the pump and probe wavelengths are selected to be 400 and 750 nm, respectively. **Figure** **2**A shows the typical transient reflectance signals (Δ*R*/*R*) under different pump fluences. The Δ*R*/*R* immediately reaches a maximum value after photoexcitation for all pump fluences. Notably, the decay rate in the first 10 ps dramatically increases under the pump fluence larger than 120 µJ cm^−2^, and an oscillation feature appears as marked by the dashed lines in Figure 2A. It is noted that high‐order recombination terms (carrier‐carrier annihilation, Auger recombination, etc.) can increase the decay rate and will complicate the analysis. To exclude these effects, the maximum Δ*R*/*R* has been extracted as a function of the pump fluence. It presents a linear relationship (Figure S5A, Supporting Information), indicating that the high‐order recombination terms can be neglected in this excitation range. The transient absorption decay dynamics (Δ*T*/*T*) are shown in Supporting Information (Figure S8, Supporting Information). The maximum Δ*T*/*T* values also linearly depend on the pump fluence, and the normalized decay dynamics are consistent throughout the entire excitation density range (Figure S8A, Supporting Information). It further confirms that the high‐order recombination terms can be neglected in this excitation range. The Δ*R*/*R* signal can be characterized by an exponentially decaying component superimposed with an oscillatory signal. The inset in Figure 2B displays the oscillatory signal after subtracting the exponentially decaying component. Figure 2B shows the corresponding fast Fourier transform (FFT) of the oscillatory components. The main recognizable feature is the only peak at 30.3 GHz, which is associated with the longitudinal acoustic (LA) phonon mode in BiFeO_3_ thin films.^[^ ^22^, ^23^, ^24^ ^]^

### 2.3 Electron‐Phonon Coupling Mediated Carrier Transport

The dynamics results illustrate that phonons can couple with photoexcited carriers and modulate the carrier dynamics in the BiFeO_3_ thin films. However, whether this electron‐phonon coupling can impact the carrier transport property is still unknown. The TAM has been previously demonstrated as an effective technique to directly image carrier transport with a high time resolution and spatial precision.^[^ ^33^ ^]^ Here, the pump and probe wavelengths are selected to be 400 and 750 nm, respectively. The setup and experimental details can be found in the Supporting Information. The pump fluence is set to be 395 µJ cm^−2^ to reach a better signal‐to‐noise ratio and avoid high‐order recombination processes.

**Figure 3:**
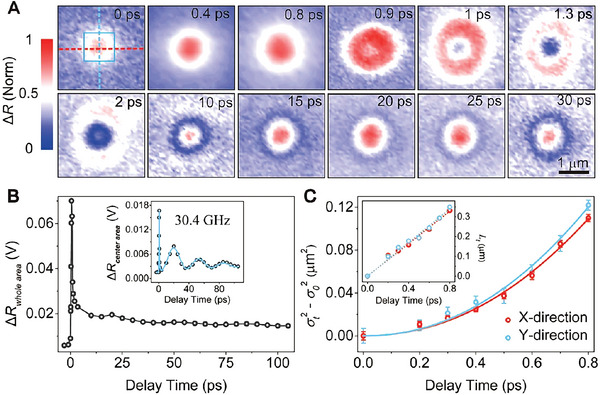
TAM images and quasi‐ballistic carrier transport. A) TAM images at different delay times as labeled. The pump and probe wavelengths are 400 and 750 nm, respectively, and the pump fluence is 395 µJ cm^−2^. B) The overall integrated population of the photogenerated carriers extracted from the TAM images as a function of pump‐probe delay times. Inset: Time evolution of the carrier population integrated from the square area as marked by the blue solid line in the TAM image. C) Time evolution of the $\sigma_{t}^{2}-\sigma_{0}^{2}$ probe along the two perpendicular directions as marked by the dash lines in the TAM image, where $\sigma_{0}^{2}$ is the width of the spatial carrier distribution obtained at zero pump‐probe delay. The blue and red lines represent fits using the equation, *Dt* ^*α*^. The diffusion exponent *α* ≈ 2 signifies that the carriers propagate in a quasi‐ballistic manner. Inset: Time evolution of the relative change in the quasi‐ballistic transport length, $L_{t}=\sqrt{\sigma_{t}^{2}-\sigma_{0}^{2}}\;$. The dotted lines represent the resultant fit of *L* = *vt*, where *v* is a coefficient that provides the velocity of the quasi‐ballistic transport.

**Figure** **3**A displays the two‐dimensional TAM images at different delay times. If a normal carrier diffusion scenario is presented in the BiFeO_3_ thin film, one should observe the carrier distribution gradually spread broader with a Gaussian distribution. However, the diameters of the Gaussian distributions expand significantly within 1 ps, and a doughnut shape carrier distribution appears at 1 ps. In addition, this feature recovers back to a Gaussian distribution at 15 ps. The unique evolution process implies that unexpected transport behavior along the radial direction exists apart from the normal diffusion scenario. Furthermore, the carrier distributions appear to be constantly oscillating which lasts up to 120 ps. Figure 3B displays the time evolution of the carrier population. Here, the Δ*R*_whole area_ and Δ*R*_center area_ signals are obtained by integrating the whole and the center area (1 µm^2^) of the TAM images, respectively. The Δ*R*_whole area_ signal decays exponentially and shows no obvious oscillation characteristics. The Δ*R*_center area_ signal, however, shows consistent decay properties as the transient reflection dynamics (Figure 2A). They both decrease rapidly in the first few picoseconds and exhibit oscillatory components in longer delay times. Note that the transient reflection dynamics (Figure 2A) can only detect the carrier population with the probe beam size. This consistency further confirms that the carrier diffusion is mediated by the LA phonon mode.

The solution to the diffusion equation with a Gaussian initial condition is given by $\mathrm{MSD}\;=\;\sigma_{t}^{2}\;-\sigma_{0}^{2}$ which is proportional to *t* ^*α*^, where MSD is the mean‐squared displacement, and *α* is the diffusion exponent (experimental details in the Supporting Information) ^[^ ^44^, ^45^ ^]^. For normal diffusion, the diffusion coefficient is independent of time and *α* equals to 1. For the superdiffusive transport scenario such as ballistic transport, the diffusivity depends on time and *α* is larger than 1. Notably, the diffusion model can only be used to fit the Gaussian distribution. When the carriers are coupled with the phonons and result in a doughnut shape distribution, this diffusion model is no longer applicable. Figure 3C displays the time evolution of MSD along the two perpendicular directions as marked by the dash lines in the TAM image (Figure 3A). Interestingly, the fittings give the diffusion exponents of *α* = 2.09 ± 0.12 and *α* = 2.11 ± 0.14 for the horizontal and vertical directions, respectively. The similar values along horizontal and vertical directions illustrate undistinguishable anisotropic diffusion behavior. The pump polarization‐dependent transient reflection dynamics also show isotropic response (Figure S6, Supporting Information). The previous study illustrates that the relative propagation direction of the light and the ferroelectric polarization can impact the photoresponse of ferroelectrics.^[^ ^46^ ^]^ However, the piezo force microscopy results show that the domain structures in BiFeO_3_ thin films are small and randomly arranged, which is one order of magnitude smaller than the probe beam size (Figure S2, Supporting Information). The small domain size and the disordered domain structures are responsible for the isotropic response. Moreover, the value (*α* = 2) signifies that the carriers propagate in a quasi‐ballistic manner in the first picosecond. The quasi‐ballistic transport length (*L_t_*) can then be plotted as $L_{t}=\;vt\;=\sqrt{\sigma_{t}^{2}-\sigma_{0}^{2}}\;$, where *v* is a coefficient that provides the velocity of the quasi‐ballistic transport (Inset in Figure 3C). The linear region gives the quasi‐ballistic velocities of 4.09 × 10^5^ and 4.24 × 10^5^ m s^−1^ for the horizontal and vertical directions, respectively. The quasi‐ballistic transport length is ≈340 nm within the first picosecond, which is even larger than that of hot electrons in Si (20 nm), GaN (14 nm), and CH_3_NH_3_PbI_3_ perovskites (230 nm).^[^ ^34^, ^35^, ^36^ ^]^ The long quasi‐ballistic transport length is significant for BiFeO_3_‐based high‐efficiency electronic device applications.

We now discuss the possible origins of such spatial propagation at room temperature. First, it is noted that the measured quasi‐ballistic velocity (4 × 10^5^ m s^−1^) is orders of magnitude larger than the velocity of phonons (typically below 10^3^ m s^−1^). This means that the pure phononic origin is not responsible for the measured Δ*R*/*R* signal. Second, previous works illustrate that the collective phase modes of the electronic condensate can hybridize with the phonon modes in an excitonic insulator.^[^ ^47^, ^48^ ^]^ The hybrid modes result in the excitations of mixed electronic and phononic nature and propagate ballistically at velocities close to the pure phase modes (on the order of 10^5^–10^6^ m s^−1^) over several picoseconds. However, such hybrid modes cannot explain the observed quasi‐ballistic transport behavior in this work due to the low carrier diffusion constant and rather flat dispersion relation of the magnon sidebands in BiFeO_3_.^[^ ^49^ ^]^ Other potential explanations, such as phonon‐polaritons and exciton‐polaritons, either propagate at velocities larger than 5 × 10^6^ m s^−1^, or require a substantially high *Q* factor to reach the picosecond lifetimes.^[^ ^48^ ^]^ The BiFeO_3_ thin films and the measured velocity of ≈4 × 10^5^ m s^−1^ do not agree with those conditions. Besides, none of these possibilities can exhibit the spatiotemporal evolutions as shown in Figure 3A.

One remaining possibility is the coupling between hot carriers and the LA phonon mode. In our results, the spatiotemporal evolutions of the Δ*R*/*R* signals reveal a propagation velocity of 4 × 10^5^ m s^−1^ within the first picosecond. Noting that the ballistic velocity of hot carriers is directly related to the excess energy above the bandgap.^[^ ^36^ ^]^ In BiFeO_3_, the electrons and holes have similar smallest effective masses and $m_{e}^{*}\approx 0.39\;m_{0}$, where *m*_0_ is the electron rest mass.^[^ ^50^ ^]^ Then, the corresponding ballistic velocity *v_max_* is given by $\Delta \;E_{e}\;=\;1/2\;m_{e}^{*}v_{max}^{2}$, where Δ *E_e_* = 3.1 − 2.66 = 0.44 eV, leading to a value of ≈5.74 ×  10^5^ m s^−1^. The measured propagation velocity (4 ×  10^5^ m s^−1^) is close to *v_max_*, indicating the hot carrier quasi‐ballistic transport. Moreover, the doughnut shape carrier distribution illustrates that the hot carriers can strongly couple to the LA phonons. Previous works illustrate that when carriers are strongly coupled with the low‐energy acoustic phonons, the hot carrier relaxation lifetime can be increased in contrast to coupling with the optical phonons.^[^ ^51^, ^52^, ^53^, ^54^ ^]^ Besides, the strong hot carrier‐LA phonon coupling may lead to carrier propagation with electronic and phononic nature.^[^ ^54^, ^55^, ^56^ ^]^ The hot carrier ballistic motion leads to a favored propagation in the radial direction, which is modulated by the combination of the ballistic driving terms and the phonon‐induced electronic motions.^[^ ^56^ ^]^ Due to the small carrier diffusion constant of BiFeO_3_ (Figure S10, Supporting Information), the carrier normal diffusion can be neglected compared to the quasi‐ballistic transport. The Gaussian population distribution evolves into a doughnut shape presenting the phononic nature. In the next step (after 5 ps), the phononic nature will drag the carriers to move toward the center spot. Then, the carrier population in the center area increases, while the carrier population around the doughnut shape area decreases. As a result, the doughnut shape evolves into the Gaussian distribution again as time goes by. This also indicates that the Δ*R*/*R* signals do not represent acoustic phonons, because the acoustic phonons will not experience such spatial evolutions and will propagate like a wave.^[^ ^57^ ^]^

Figure S12 (Supporting Information) displays the carrier transport properties after 10 ps. Distinct periodic oscillations can be observed in the time evolutions of the variances $\sigma_{t}^{2}$ (Figure S12B, Supporting Information). The oscillation frequency of ≈30.5 GHz is consistent with the oscillation frequency of the LA phonon mode (Figure 2B). This suggests that coupling between the carriers and the LA phonon mode will result in the periodic change of the carrier density distribution. The time derivative over the oscillatory variances then gives the time‐dependent carrier diffusion coefficient *D_t_* mediated by the electron‐phonon coupling (Figure S12C, Supporting Information). The maximum mediated diffusion coefficient can reach 45 cm^2^ s^−1^, which is still two orders of magnitude larger than the bulk (0.16 cm^2^ s^−1^) measured by the TAM (Figure S11, Supporting Information). The increased diffusion coefficient has strong implications for their optoelectronic applications and implies new ways to modulate the carrier transport in BiFeO_3_ thin films.

## 3 Discussion

**Figure 4:**
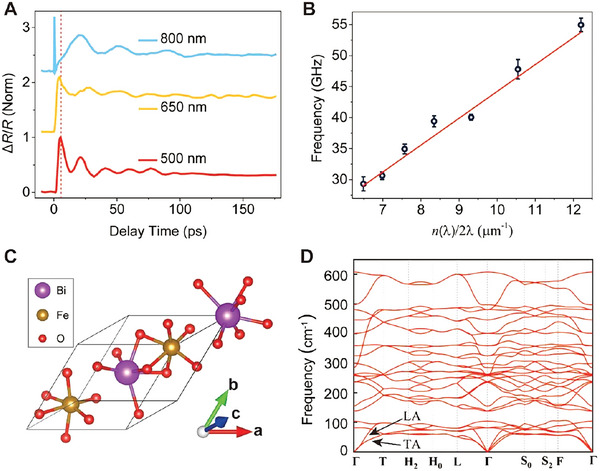
Transient phonon characteristics and phonon dispersion analysis. A) Transient reflection dynamics with different probe wavelengths. The pump wavelength is 400 nm and the intensity is 276.5 µJ cm^−2^. The red dash line marked out the oscillation phase shifts. B) Probe wave vector dependence of the oscillation frequencies fitted from the oscillation components shown in (A). The red solid line gives the sound velocities of the acoustic phonon mode. *n*(*λ*) is the wavelength‐dependent refractive index. C) Schematic of the R3c phase BiFeO_3_ primitive unit cell. D) Calculated phonon dispersion curves for BiFeO_3_ along high symmetry directions from first‐principle DFT‐based approaches.

Our results indicate that the electron‐phonon coupling can strongly mediate the carrier dynamics and transport properties in BiFeO_3_ thin films. It is important to identify the exact phonon mode that dominates in the coupling. Due to the phonon dispersion relation, the coupling between photoexcited carriers and the acoustic phonon mode with different oscillation frequencies is expected when the probe wavelength is changed.^[^ ^58^ ^]^ **Figure** **4**A displays the Δ*R*/*R* signals detected with different probe wavelengths. Two distinguishing features are presented. First, the oscillation period changes with the probe wavelength. The corresponding oscillation frequencies are extracted in Figure 4B. The linear fit gives the sound velocity of the phonon mode as 4.32 ± 0.37 km s^−1^.^[^ ^58^ ^]^ Second, the cosine‐like oscillations give the initial phases at the delay time near zero (the dashed line in Figure 4A), which are always close to either 0 or *π*. In multiferroics, the photoexcited carriers can lead to the instantaneous change of the quasi‐equilibrium positions of the positive and negative charges due to the inverse piezoelectric effect. This will result in the generation of coherent acoustic phonons, which is called the displacive excitation of coherent acoustic phonons.^[^ ^47^ ^]^ The initial phases of the cosine‐like oscillations agree with the displacive behaviors of the lattice, indicating that the inverse piezoelectric effect is responsible for the generation of the observed acoustic phonons.^[^ ^58^, ^59^ ^]^

To further verify the origin of the observed phonon mode, the first‐principles calculations based on density functional theory (DFT) are performed using the Vienna ab initio simulation package (VASP) code.^[^ ^60^ ^]^ The primitive unit cell of R3c BiFeO_3_ containing 10 atoms is adopted and G‐type antiferromagnetism is preserved. The projector augmented‐wave (PAW) approximation and Perdew‐Burke‐Ernzerhof (PBE) exchange‐correlation function is used to consider the electron‐nuclear interactions and electron‐electron interactions.^[^ ^61^, ^62^ ^]^ A typical effective Hubbard U value of 4 eV is applied for 3d electron of Fe atoms.^[^ ^63^ ^]^ The energy cutoff is set to 650 eV. The convergence criteria for energy and atomic forces are smaller than 10^−8^ eV and 0.001 eV Å^−1^, respectively. To obtain the vibrational properties, real‐space force constants are calculated using the finite displacement method implemented in the VASP code, and phonon frequencies are calculated by PHONONPY code^[^ ^64^ ^]^ using 3 × 3 × 3 supercell with 3 × 3 × 3 Monkhorst‐Pack k‐point mesh. Figure 4D presents the calculated phonon dispersion curves along Γ(0,0,0)‐T(0.5,0.5,0.5)‐H_2_‐H_0_‐L(0.5,0,0)‐Γ‐S_0_‐S_2_‐F(0,5,0,0.5)‐Γ directions. It shows that three acoustic phonon modes exit along the Γ‐T direction, with two lower degenerated transverse acoustic (TA) phonon modes and one higher LA phonon mode. Furthermore, according to *v_g_* = *dω*/*dq* , the group velocities of the LA and TA modes near the Γ point are calculated as 4.38 and 1.92 km s^−1^, respectively. The calculated group velocity of the LA mode is in good agreement with the measured sound velocity of 4.32 ± 0.37 km s^−1^. Thus, it can be confirmed that the observed phonon mode corresponds to the LA phonon mode, and it polarizes and propagates along the [111] crystallographic direction.

The dynamics and transport properties are also investigated in (100) and (110) BiFeO_3_ epitaxial thin films. The dynamics and corresponding FFT results are shown in the Supporting Information (Figure S13, Supporting Information). Both the dynamics in (100) and (110) BiFeO_3_ thin films show clear oscillatory components, and the extracted oscillation frequencies are ≈29.21 and 31.02 GHz, respectively. These frequencies are consistent with the LA phonon mode found in (111) BiFeO_3_ thin films, which further indicates that photoexcited carriers will couple with this LA phonon mode in BiFeO_3_. Moreover, the carrier diffusion coefficient is also enhanced in the initial 1 ps (Figure S14, Supporting Information). Notably, it is observed that the enhancement is smaller in (100) and (110) than that in (111) BiFeO_3_ thin films. In transient reflection spectroscopy, the effective probing depth is near the surface.^[^ ^65^ ^]^ Thus, the observed carrier diffusion can be considered as the carriers near the surface plane, which are different orientations in these three epitaxial thin films. Since the observed LA phonon mode is polarized and propagated in the (111) plane, the enhancement of this coupling on carrier diffusion should be the most obvious in the (111) plane.

In summary, we have directly observed the impact of electron‐phonon coupling on carrier transport in BiFeO_3_ epitaxial thin films. The carrier transport behavior is significantly altered by coupling to the LA phonons, resulting in a temporal doughnut shape carrier distribution. The hot carriers can couple to the LA phonons and quasi‐ballistic transport with a velocity of ≈4.24 × 10^5^ m s^−1^, which gives a transport length of 340 nm within the first picosecond. After the cooling processes, the maximum carrier diffusion coefficient can still reach 45 cm^2^ s^−1^ mediated by electron‐phonon coupling, which is over two orders of magnitude larger than that in bulk BiFeO_3_. The high carrier mobility and transport length could be the key factor for efficient charge transport and electronic device applications. Besides, our approach provides a compelling method to study the carrier decay dynamics and transport properties in a variety of materials with electron–phonon interactions.

## 4 Experimental Section

### Sample Preparations and Characterizations

The epitaxial thin films were deposited by pulsed laser deposition, the ceramic target of Sr_3_Al_2_O_6_ was prepared by solid‐state reaction method,^[^ ^37^, ^38^, ^39^ ^]^ and the ceramic targets of BiFeO_3_ and SrRuO_3_ were commercially available. Sr_3_Al_2_O_6_, SrRuO_3,_ and BiFeO_3_ thin films were deposited sequentially on SrTiO_3_ substrates using a KrF excimer laser with a wavelength of 248 nm. The Sr_3_Al_2_O_6_ layer was grown at 750 °C under an oxygen pressure of 15 Pa, the energy density was ≈0.8 J cm^−2^ and the repetition rate was 3 Hz. The SrRuO_3_ layer was grown at 650 °C under an oxygen pressure of 20 Pa, the energy density was ≈0.8 J cm^−2^ and the repetition rate was 3 Hz. The BiFeO_3_ layer was grown at 600 °C under an oxygen pressure of 1 Pa, the energy density was ≈0.7 J cm^−2^ and the repetition rate was 5 Hz.

High‐resolution X‐ray diffraction measurements (*θ*‐2*θ* scan) were conducted by a PANalytical Empyrean diffractometer. The surface morphology was characterized by atomic force microscopy (Bruker Icon). Atomic‐resolution high‐angle annular dark‐field (HAADF) and annular bright‐field (ABF) images were obtained on a JEOL ARM200F microscope with CS‐corrected STEM operated at 200 kV.

### Transient Absorption Microscopy

An optical parametric amplification (ORPHEUS twins, Light Conversion) was pumped by the output of a high‐repetition‐rate amplifier (repetition rate of 400 kHz, 1030 nm central wavelength, and pulse duration of ≈120 fs, PHAROS, Light Conversion) to generate two independent wavelength‐tunable femtosecond lasers. One as a 400 nm pulse was used to pump the BiFeO_3_ thin film, and the other one was tuned from 500 to 800 nm and acted as the probe light. A mechanical translation stage (DDS600‐E/M, Thorlabs) was used to delay the probe to the pump. Both the pump and probe beams were spatially filtered before being focused onto the sample by a 60× objective (0.95 NA, Nikon). The probe beam was collected by the same objective (or by another 50× objective in the transient absorption scheme) and filtered by a 458 nm long‐pass filter before being detected by an avalanche photodiode (APD410A/M, Thorlabs). The pump‐induced change of the probe was detected by a lock‐in amplifier (Signal Recovery 7265, Ametek SI). A pair of galvanometer mirrors (Thorlabs GVS002) was used to scan the probe beam relative to the pump in space to obtain the carrier population distribution profiles. The probe beam profile did not vary when scanning the entire optical delay stage. The schematic of the TAM setup is presented in the Supporting Information.

## Conflict of Interest

The authors declare no conflict of interest.

## Author contributions

Z.O. and B.P. contributed equally to this work. Z.O., T.W., and X.H. designed the experiments. Z.O. carried out the optical measurements and data analysis with help from Z.L., C.W., Y.Z., and H.C. W.C., Q.W., and L.Z. carried out the calculation. R.Q., B.P., M.L., and Z.H. designed and provided the materials to study. G.D. carried out the TEM study. Y.W., L.M., T.L., and T.Y. carried out the PFM study. Z.O., T.W., and Z.L. wrote the manuscript. All authors edited the manuscript.

## Supporting information

Supporting Information

## Data Availability

The data that support the findings of this study are available in the supplementary material of this article.
