# Supplementary material for: Strong Electron‐Phonon Coupling Mediates Carrier Transport in BiFeO3
Source: Adv Sci (Weinh). 2023 May 23;10(22):2301057. doi: 10.1002/advs.202301057 (PMC10401121; doi:10.1002/advs.202301057)
Supplement: Supplementary file 1 — Supporting Information [file ADVS-10-2301057-s001.pdf]

ADVANCED SCIENCE
Open Access

Supporting Information

for *Adv. Sci.*, DOI 10.1002/advs.202301057

Strong Electron-Phonon Coupling Mediates Carrier Transport in $BiFeO_3$

Zhenwei Ou, Bin Peng, Weibin Chu, Zhe Li, Cheng Wang, Yan Zeng, Hongyi Chen, Qiuyu Wang, Guohua Dong, Yongyi Wu, Ruibin Qiu, Li Ma, Lili Zhang, Xiaoze Liu, Tao Li, Ting Yu, Zhongqiang Hu, Ti Wang*, Ming Liu and Hongxing Xu**

Supporting Information

Strong electron-phonon coupling mediates carrier transport in $BiFeO_3$

Zhenwei Ou, Bin Peng, Weibin Chu, Zhe Li, Cheng Wang, Yan Zeng, Hongyi Chen, Qiuyu Wang, Guohua Dong, Yongyi Wu, Ruibin Qiu, Li Ma, Lili Zhang, Xiaoze Liu, Tao Li, Ting Yu, Zhongqiang Hu,* Ti Wang,* Ming Liu, and Hongxing Xu*

*Corresponding author. zhongqianghu@xjtu.edu.cn (Zhongqiang Hu); wangti@whu.edu.cn (Ti Wang); hxxu@whu.edu.cn (Hongxing Xu)

1. Basic principles of transient absorption microscopy (TAM)

In pump-probe experiments, the pump pulse excites the sample into the excited states, and the delayed probe pulse monitors the carrier population. By scanning the pump and probe beams relative to each other and recording the corresponding relative changes in probe transmission or reflection, the time-dependent carrier and exciton density images are generated. These images can be used to monitor the carrier transport.

The solution to the diffusion equation with a Gaussian initial condition is given by MSD $= \sigma_t^2 - \sigma_0^2$, which is proportional to t^α, where MSD is the mean-squared displacement, and α is the diffusion exponent [1-2]. For normal diffusion, the diffusion coefficient is independent of time and α equals to 1.

At 0 ps, the TAM image represents the initial photo-generated carrier population created by the pump pulse.

$$n(x,0) = Nexp[-\frac{(x-x_0)^2}{2\sigma_0^2}] \quad (1)$$

At later delay times, the TAM curves reflect carrier diffusion away from the initial excitation volume. If the carrier transport is diffusive, the time and spatial dependent carrier density is given by,

$$\frac{\partial n(x,t)}{\partial t} = D\left[\frac{\partial^2 n(x,t)}{\partial x^2}\right] - \frac{n(x,t)}{\tau} \quad (2)$$

where $n(x,t)$ is the carrier population as a function of position and time, D is the diffusion coefficient, and τ is the carrier lifetime.

The solution to equation (2) dictates that the population follows a Gaussian distribution at any later delay time *t* given by,

$$n(x,t) = Nexp[-\frac{(x-x_0)^2}{2\sigma_t^2}] \quad (3)$$

The TAM profiles are fitted by one-dimensional Gaussian functions with variances of σ_t^2, where the σ_t^2 is the time-dependent variance of the Gaussian profiles at delay time *t*. The diffusion constant D is then given by $D = \frac{\sigma_t^2 - \sigma_{t0}^2}{2t}$, and the average diffusion distance L that excitons or charge travels in time *t* is given by $L_t^2 = \sigma_t^2 - \sigma_0^2$.

2. Sample Characterization

Surface morphologies and ferroelectricity characterizations：
To confirm the quality of the growth thin films, the atomic force microscopy (AFM) topographies of (100), (110), and (111)-oriented $BiFeO_3$ were measured (Figure S1). The morphologies show relatively uniform crystallinities and smooth surfaces. The average roughness (R_a) and the square root roughness (R_q) for the (100), (110), (111)-oriented $BiFeO_3$ epitaxial thin films are 2.2 and 3.5 nm, 1.85 and 5.25 nm, 1.11 and 1.41 nm, respectively. The ferroelectric property and domain structures of the (111)-oriented $BiFeO_3$ film were also characterized using piezo response force microscopy (PFM) (Figure S2). The PFM measurements were carried out in a glovebox under an argon atmosphere at room temperature and ambient pressure (Icon, Bruker). A platinum/iridium (Pt/Ir) coated Si tip (SCM-PIT-V2) was used for PFM measurements. The amplitude and phase hysteresis loops confirm the ferroelectricity of the $BiFeO_3$ films. The in-plane (IP) piezo response force microscopy (IP-PFM) images, however, show that the domain structures are small (smaller than 50 nm) and randomly arranged. In our setup configuration, the pump and probe beams are about 1 μm, which is much larger than the domain size. The small domain area and the disordered domain structures are the main reasons for the isotropic response of the transient dynamics. The conventional X-ray diffraction and reciprocal space mapping were also measured to confirm the single-crystallinity and epitaxial growth (Figure S3). The sharp peaks (FWHM $\leq 0.11°$) of the rocking curves (Figure S3B) indicate the good single-crystalline properties of the $BiFeO_3$ thin films. The diffraction peaks of the $BiFeO_3$ layers follow that of the $SrTiO_3$ in both conventional X-ray diffractions (Figure S3A) and reciprocal space mappings (Figure S3C-E). Therefore, the $BiFeO_3$ thin films have good epitaxy stack structures and single crystalline. The small peaks apart from the diffraction peaks of $BiFeO_3$ and $SrTiO_3$ in the $\theta - 2\theta$ scans and RSM images arise from the diffractions of unfiltered $WL\alpha1$ and $WL\alpha2$ X-rays. The elongated diffraction spots of the RSM images arise from the stripe-like X-ray beam.

Optical characterization：
The absorption and photoluminescence (PL) were also measured using home-built microscopy (Figure S4). For better understanding, the band structure is also marked out by blue and red-shaded areas according to results from previous works [3-5]. The spectra show a sharp increase in absorbance and a strong peak in photoluminescence at about 2.66 eV. This indicates that the bandgap is about 2.66 eV, which is consistent with previous experimental results. [3] However, the absorptance onset occurs at a much lower energy of about 1.65 eV. In addition, the absorptance spectrum exhibits a broad shoulder centered at about 2 eV and a small absorbance feature below 1.5 eV. These absorption bands can be assigned to the ${}^6A_{1g} \rightarrow {}^4T_{1g}$ (below 1.5 eV) and ${}^6A_{1g} \rightarrow {}^4T_{2g}$ (between 1.65 and 2.2 eV) transitions of the Fe^{3+} ions [4-5]. These broad absorption bands are named magnon sidebands and are associated with the reduced symmetry in $BiFeO_3$ [4-5]. The small PL peak at about 1.65 eV can be attributed to the recombination of carriers in magnon sidebands.

3. Transient properties of photoexcited carriers probed in (100) and (110) planes

To further elucidate the role of electron-phonon coupling in the transport properties, the decay dynamics and transport properties are investigated in (100) and (110) $BiFeO_3$ thin films. The decay dynamics and corresponding FFT results are shown in Figure S13. Both the carrier decay dynamics in (100) and (110) $BiFeO_3$ thin films show clear oscillatory components, and the oscillation frequencies are 29.21 and 31.02 GHz, respectively. The frequencies are consistent with the LA phonon mode probed in (111) $BiFeO_3$ thin films, indicating that carriers couple with the same LA phonon mode in these thin films.

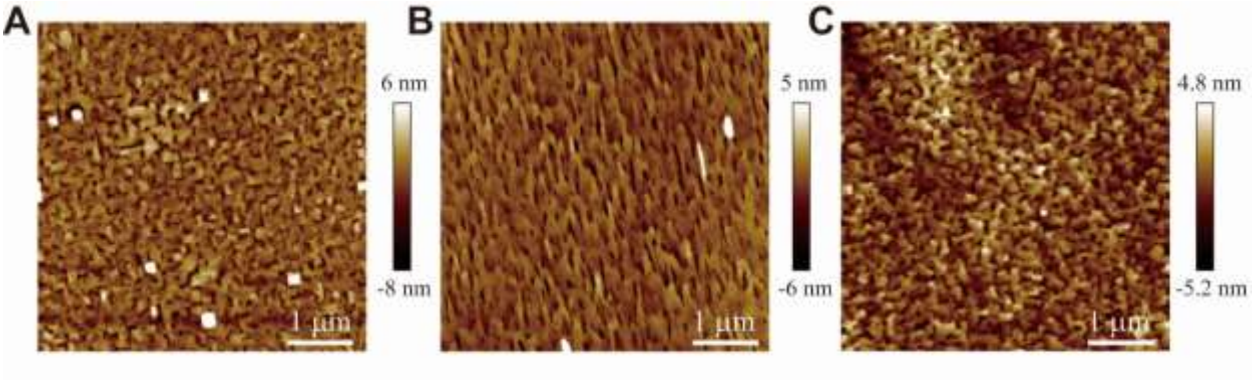

Figure S1. Sample characterization. Surface morphologies of the (100), (110), (111)-oriented $BiFeO_3$ epitaxial thin films by atomic force microscopy. The average roughness (R_a) and the square root roughness (R_q) for the (100), (110), (111)-oriented $BiFeO_3$ epitaxial thin films are 2.2 and 3.5 nm, 1.85 and 5.25 nm, 1.11 and 1.41 nm, respectively.

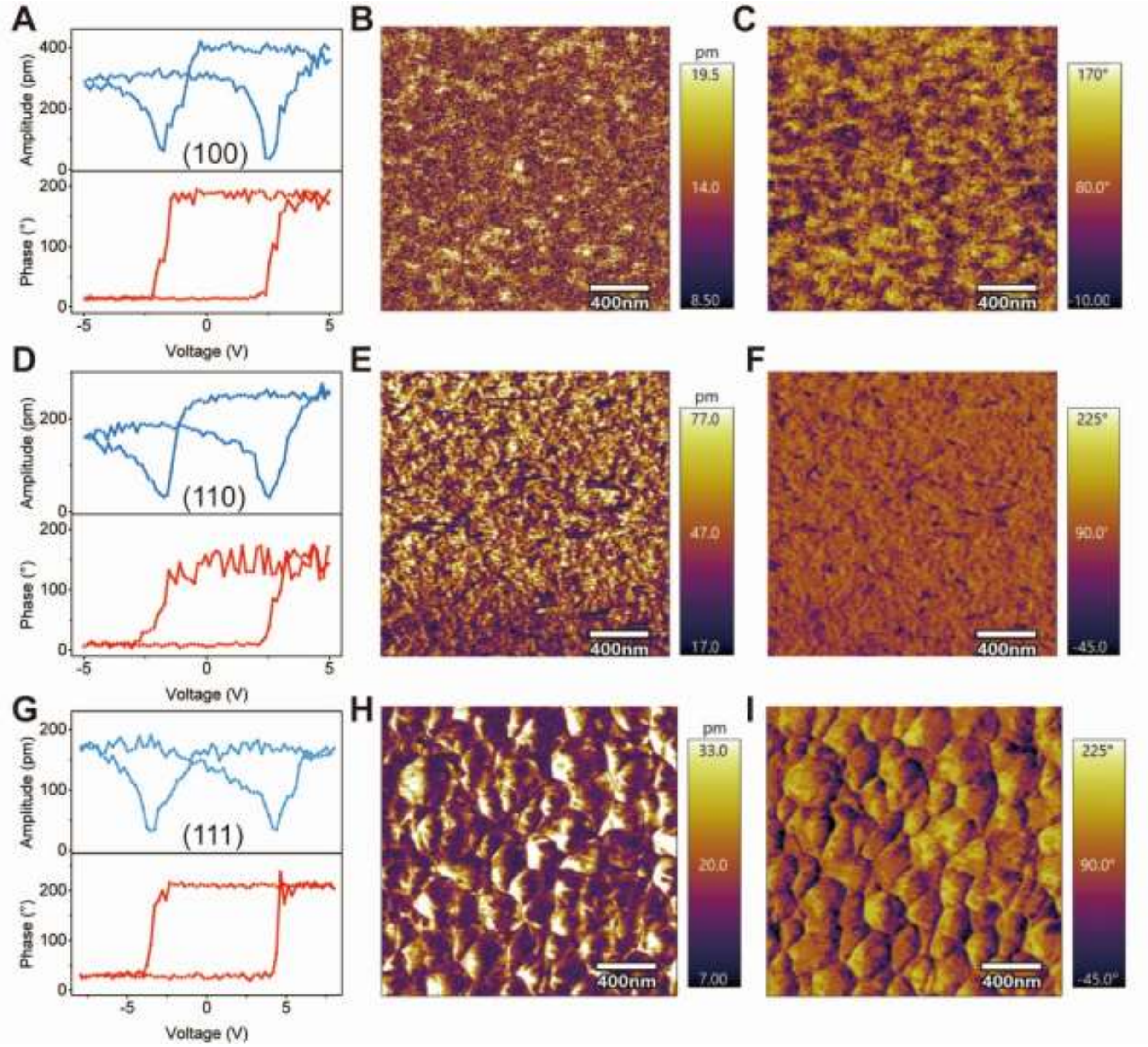

Figure S2. Ferroelectricity of $BiFeO_3$ epitaxial thin films. (A-C) Typical hysteresis loops, amplitude and phase responses of in-plane piezo force microscopy (IP-PFM) images of the (100)-oriented $BiFeO_3$ thin film, respectively. (D-F) Typical hysteresis loops, amplitude, and phase responses of IP-PFM images of the (110)-oriented $BiFeO_3$ thin film, respectively. (G-I) Typical hysteresis loops, amplitude, and phase responses of IP-PFM images of the (111)-oriented $BiFeO_3$ thin film, respectively.

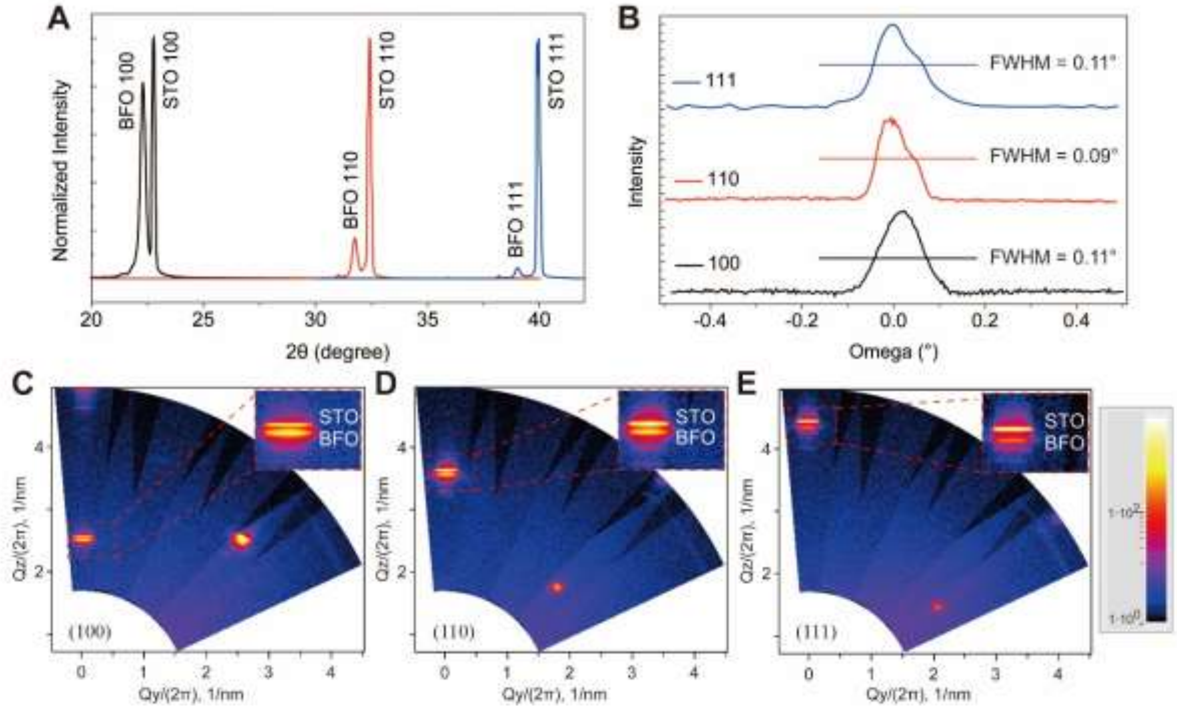

Figure S3. XRD measurement results. (A) X-ray diffraction patterns of as-grown $BiFeO_3$ epitaxial thin films. (B) Rocking curves obtained on the $BiFeO_3$ diffraction peaks. (C-E) Reciprocal space mapping (RSM) images of as-grown $BiFeO_3$ films. The small peaks apart from the diffraction peaks of $BiFeO_3$ and $SrTiO_3$ in the $\theta - 2\theta$ scans and RSM images arise from the diffractions of unfiltered WLα1 and WLα2 X-rays.

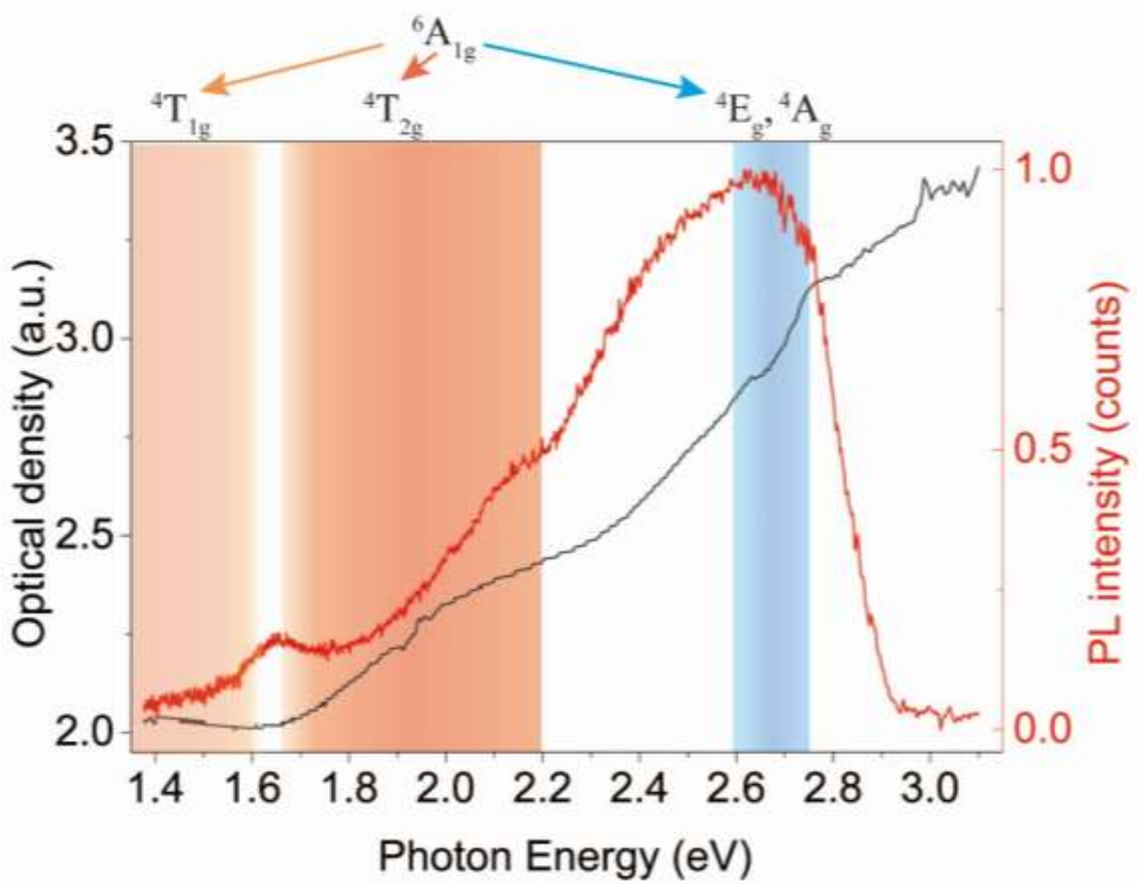

Figure S4. Steady-state absorption and PL spectra. Steady-state absorption and PL spectra. Absorption (black line), and PL spectra (red line) of (111)-oriented $BiFeO_3$ thin film at room temperature. The three light-red-shaded areas marked out the electronic-level diagram of the Fe^{3+} in $BiFeO_3$ derived from previous works.

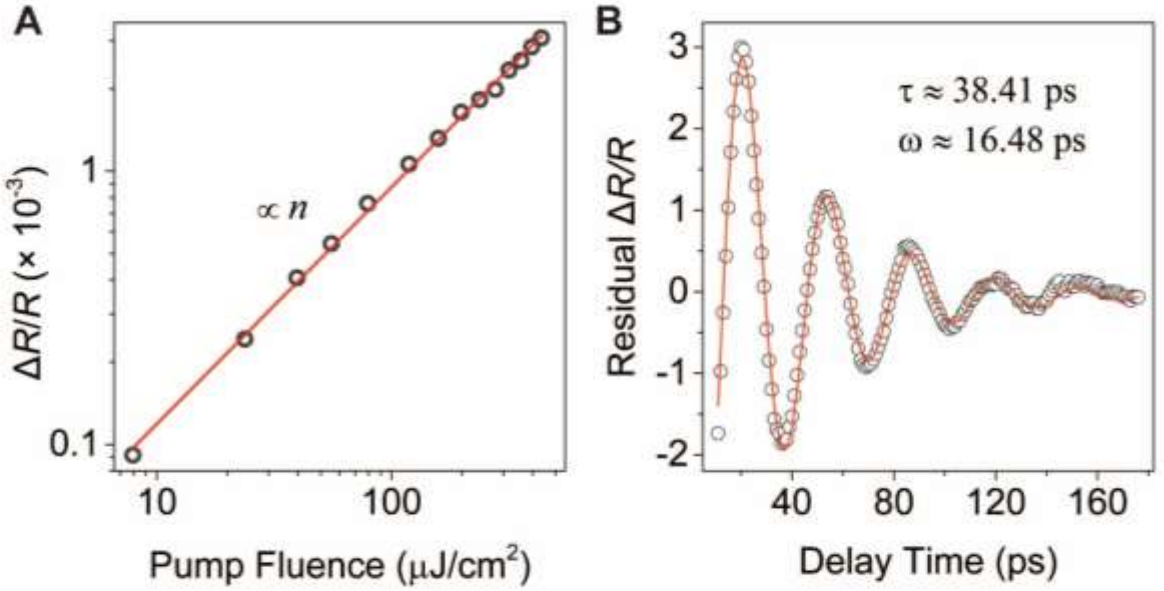

Figure S5. Transient reflection dynamics. (A) $\Delta R/R$ measured as a function of pump fluence taken at the maximum value. The solid lines represent linear fits. The pump and probe wavelengths are 400 and 750 nm, respectively. (B) The oscillatory signal in the transient reflection after subtracting the exponentially decaying component. The solid line represents fit by a sine function that decays exponentially $\Delta R/R \propto e^{-t/\tau}\sin(\pi t/\omega + \varphi_0)$, where τ is the lifetime, ω and φ_0 are the angular frequency and initial phase of the oscillation component, respectively.

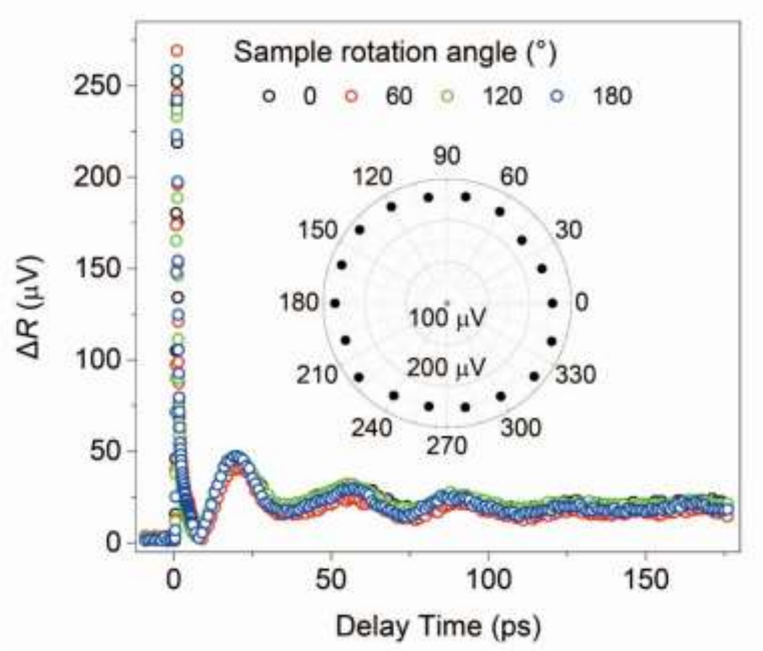

Figure S6. Pump polarization-dependent transient reflection dynamics. Pump polarization-dependent transient reflection spectra of photogenerated carriers probed in (111) plane $BiFeO_3$ thin films. To ensure the same excitation conditions, the polarization of the pump and probe lights are fixed in one direction, and the polarization dependence measurements are done by rotating the samples instead. Inset: Pump polarization dependence of the ΔR (at maximum value). The pump and probe wavelengths are 400 and 750 nm, respectively, and the pump density is 395 $\mu J/cm^2$. The measured ΔR signals show negligible differences throughout the entire range of rotation angles, which indicates that the ultrafast response is isotropic in our samples. The small domain area and the disordered property of the domain structures are the main reasons for the isotropic response of the transient dynamics.

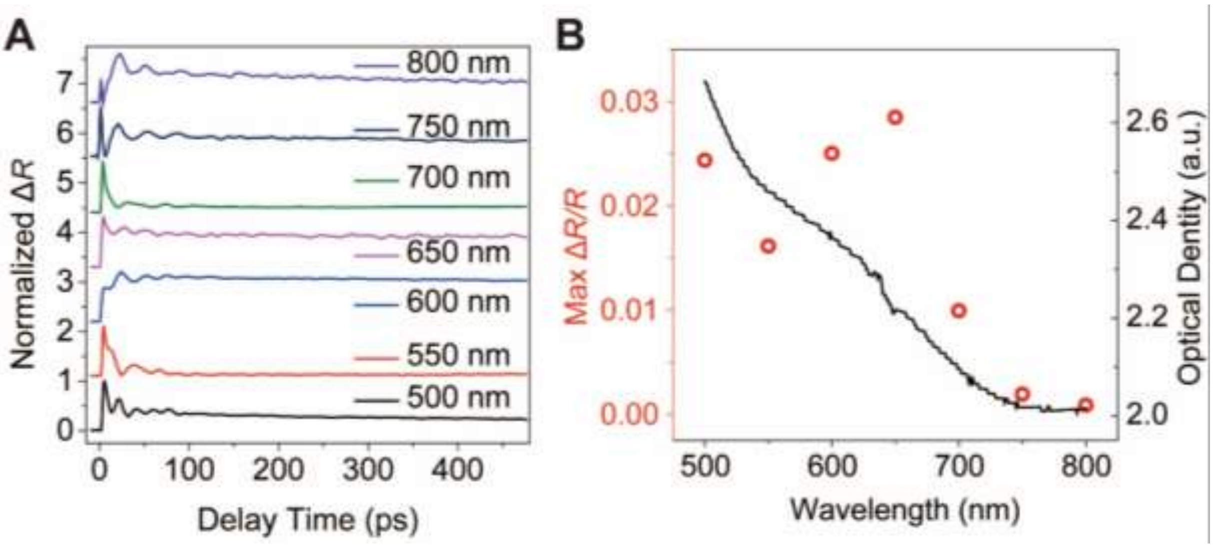

Figure S7. Probe wavelength-dependent carrier decay dynamics. (A) Probe wavelength dependent carrier decay dynamics of the (111)-oriented $BiFeO_3$ epitaxial thin film. (B) The maximum $\Delta R/R$ signals extracted from (A) in comparison with the absorption spectrum. The pump wavelength is 400 nm, and the density is 395 μJ/cm^2.

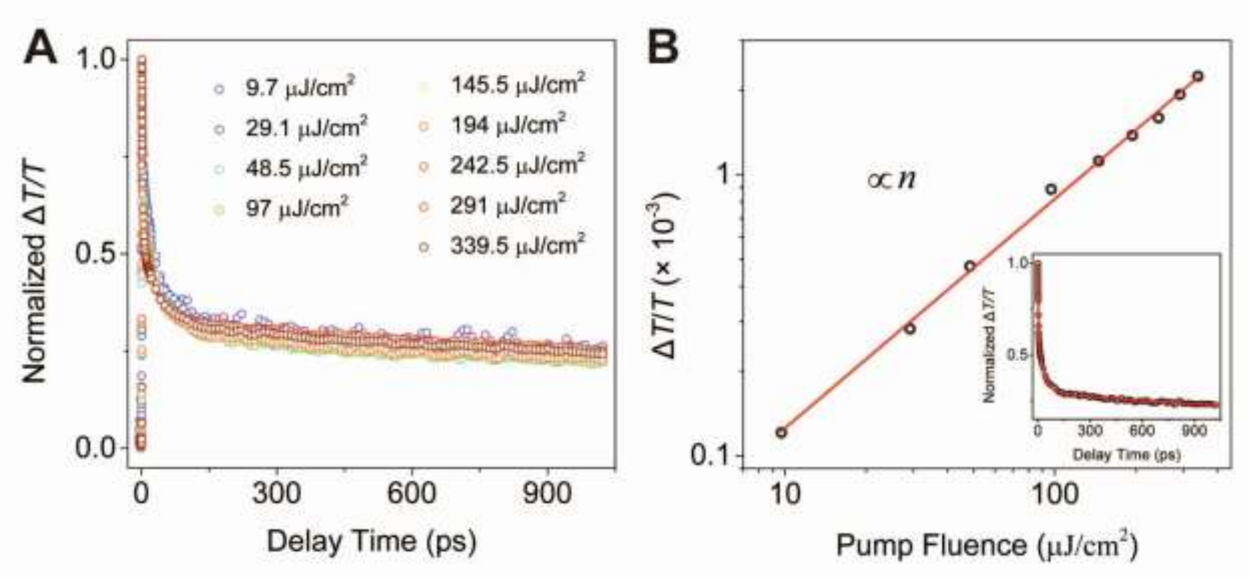

Figure S8. Transient absorption dynamics. (A) Transient absorption dynamics taken under different pump fluence. The pump and probe wavelengths are 400 and 750 nm, respectively. The normalized decay dynamics with different pump fluences exhibit high consistency. (B) The excitation density dependence of $\Delta T/T$ (taken at maximum value). The maximum $\Delta T/T$ signals present linear dependence throughout the entire range of excitation densities, indicating that the high-order recombination terms (carrier-carrier annihilation, Auger recombination, etc.) are negligible in our experimental results.

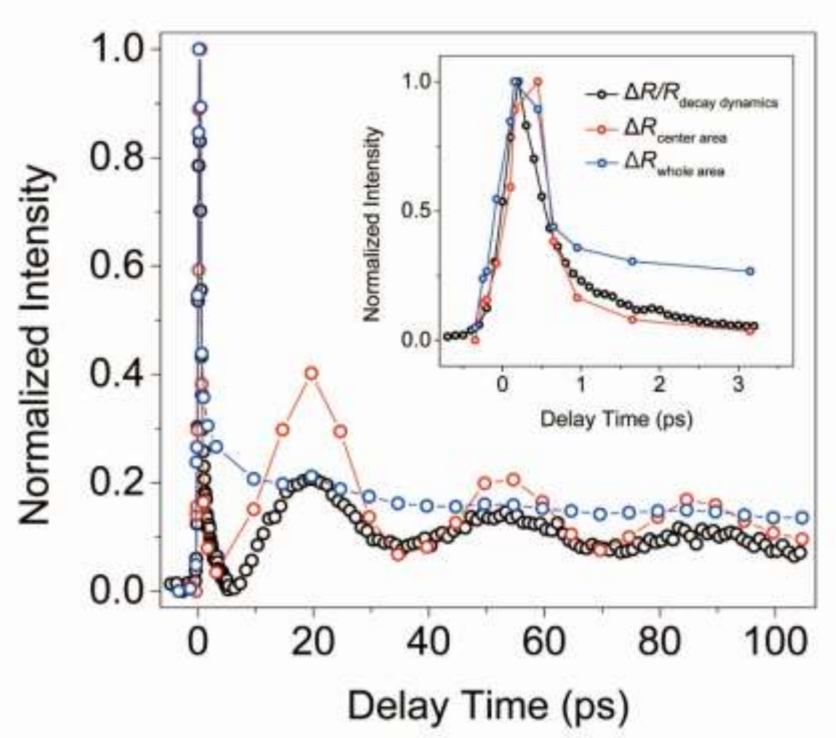

Figure S9. Carriers decay dynamics. Carrier decay dynamics of the (111)-oriented $BiFeO_3$ epitaxial thin film measured under the reflection configuration. The pump and probe wavelengths are 400 and 750 nm, respectively, and the density is 395 μJ/cm^2. The $\Delta R_{\text{whole area}}$ and $\Delta R_{\text{center area}}$ signals are obtained by integrating the overall intensity of the whole and the center (1 μm^2, similar to the size of the probe beam) areas of the TAM images, respectively. The $\Delta R/R_{\text{decay dynamics}}$ is the transient reflection dynamics. The $\Delta R/R_{\text{decay dynamics}}$ signal is consistent with the $\Delta R_{\text{center area}}$ signal. The inset shows the zoom-in decay dynamics within the first few picoseconds.

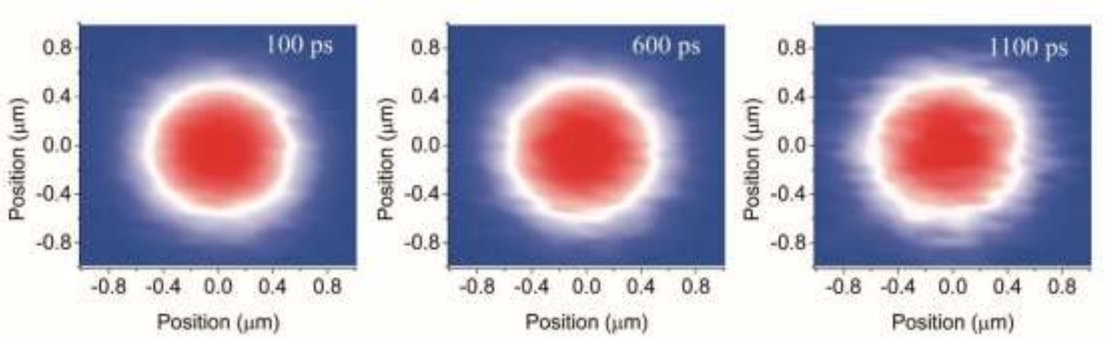

Figure S10. TAM images of the carriers in (111) plane $BiFeO_3$ thin film. The TAM images of the carriers in the (111) plane probed in the transient absorption scheme. The pump and probe wavelengths are 400 and 750 nm, respectively. The pump density is 242.5 μJ/cm^2. The TAM images show no anisotropy diffusion.

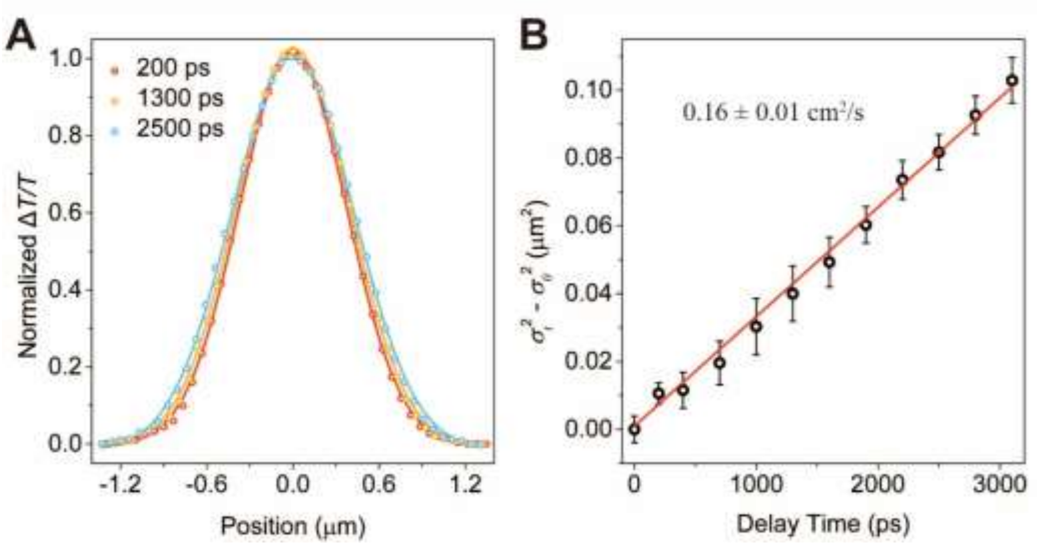

Figure S11. Carrier diffusion in (111)-oriented $BiFeO_3$ thin film measured in the transient absorption scheme. (A) The Carrier population profiles fitted with Gaussian functions at different delay times with the maximum ΔT signal normalized to unity. The pump and probe wavelengths are 400 and 750 nm, respectively. The pump density is 242.5 μJ/cm^2. (B) $\sigma_t^2 - \sigma_0^2$ as a function of pump-probe delay time. The linear fit gives the carrier diffusion constant of (111) $BiFeO_3$ thin film.

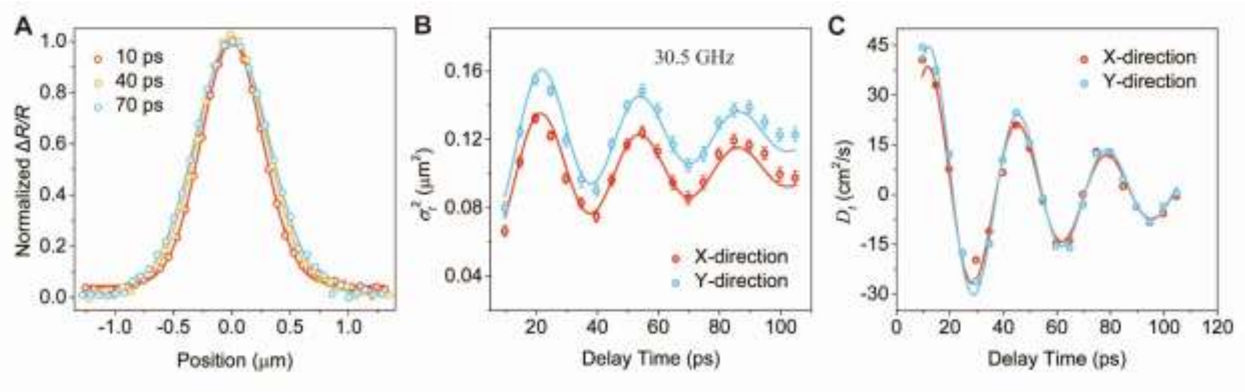

Figure S12. Carrier diffusion in (111)-oriented $BiFeO_3$ thin film measured in the transient reflection scheme. (A) Carrier population profiles fitted with Gaussian functions at three delay times with the normalized maximum Δ*R/R* signal. The pump and probe wavelengths are 400 and 750 nm, respectively. (B) σ_t^2 as a function of pump-probe delay time. (C) The time-dependent diffusion coefficient of the carriers obtained from the first-order derivation of (B). The solid lines in (B) and (C) represent the fitting by a sine function which exponentially decays $e^{-t/\tau}\sin(\pi t/\omega + \varphi_0)$, where τ is the lifetime, ω and φ_0 are the angular frequency and initial phase of the oscillation component, respectively.

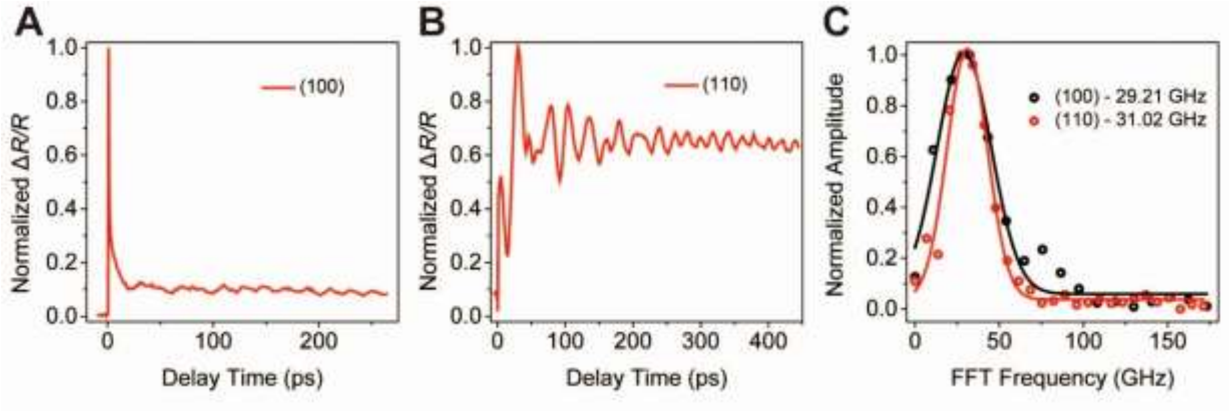

Figure S13. Transient reflection spectra of photogenerated carriers probed in (100) and (110) plane $BiFeO_3$ thin films. (A, B) Typical transient reflection signals probed in (100) and (110) planes, respectively. The pump and probe wavelengths are 400 nm and 750 nm, respectively. The pump density is 395 μJ/cm^2. (C) The FFT spectra extracted from (A) and (B).

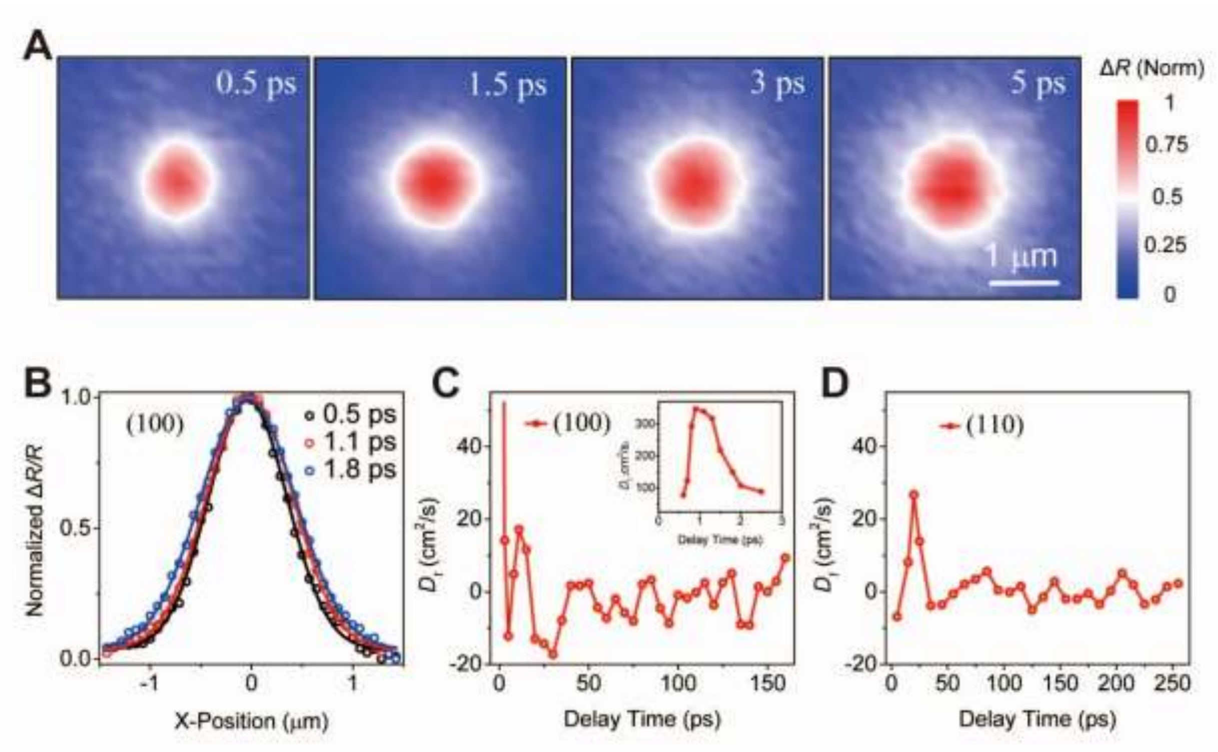

Figure S14. TAM images of the carrier transport properties probed in (100) and (110) plane $BiFeO_3$ thin films. (A) TAM images probed in (100) plane, The pump and probe wavelengths are 400 and 750 nm, respectively. The pump density is 395 μJ/cm^2. (B) One-dimensional TAM image profiles probed at 0.5, 1.1, and 1.8 ps, respectively. (C, D) Time-dependent diffusion coefficients plotted as a function of delay time in (100) and (110) $BiFeO_3$ thin films, respectively.

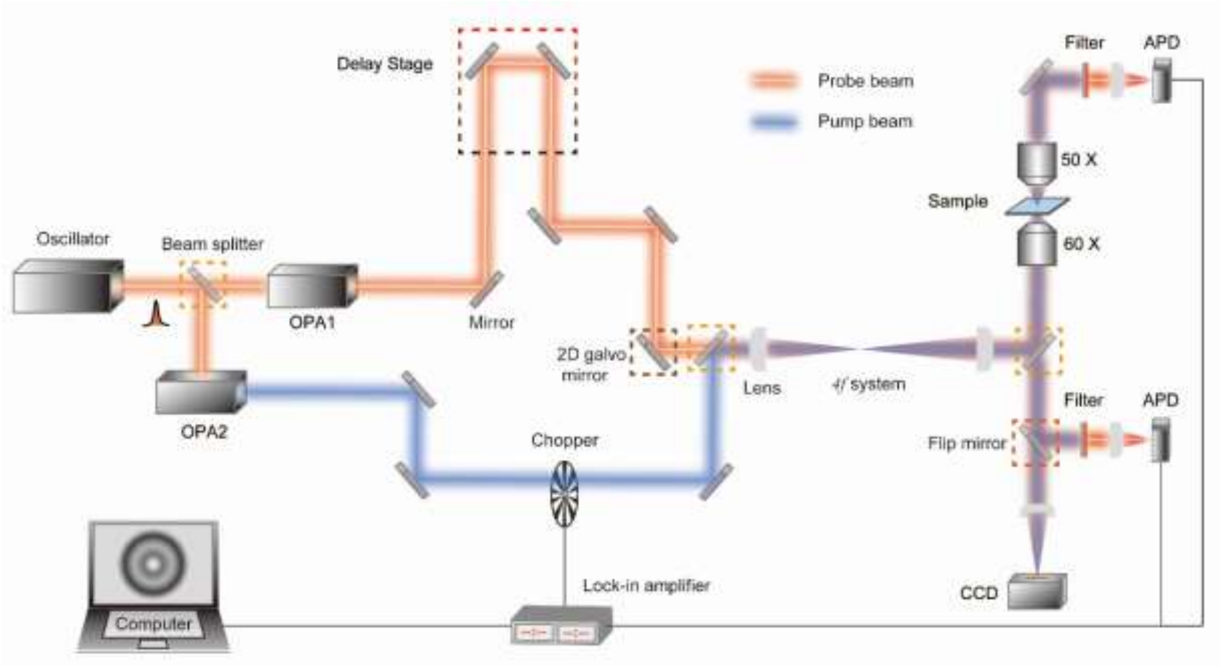

Figure S15. Experiment setup of the transient reflection/absorption microscopy. The 1030 nm laser is directed into two OPAs separately to generate two individual wavelength-tunable lights that are used as the pump and probe beams. The time delay of the pump and probe beams is modulated through the optical delay line. The probe beam is scanned by the 2D-galvo mirror to construct transient absorption images. Abbreviations: OPA, optical parametric amplifier; APD, avalanche photodiode.

References:

[1] G. M. Akselrod, P. B. Deotare, N. J. Thompson, J. Lee, W. A. Tisdale, M. A. Baldo, V. M. Menon, V. Bulović, *Nat. Commun.* **2014**, 5, 3646.
[2] J. Sung, C. Schnedermann, L. Ni, A. Sadhanala, R. Y. S. Chen, C. Cho, L. Priest, J. M. Lim, H.-K. Kim, B. Monserrat, P. Kukura, A. Rao, *Nat. Phys.* **2020**, 16, 171.
[3] D. Sando, C. Carretero, M. N. Grisolia, A. Barthelemy, V. Nagarajan, M. Bibes, *Adv. Opt. Mater.* **2018**, 6, 1700836.
[4] X. S. Xu, T. V. Brinzari, S. Lee, Y. H. Chu, L. W. Martin, A. Kumar, S. McGill, R. C. Rai, R. Ramesh, V. Gopalan, S. W. Cheong, J. L. Musfeldt, *Phys. Rev. B* **2009**, 79, 134425.
[5] M. O. Ramirez, A. Kumar, S. A. Denev, N. J. Podraza, X. S. Xu, R. C. Rai, Y. H. Chu, J. Seidel, L. W. Martin, S. Y. Yang, E. Saiz, J. F. Ihlefeld, S. Lee, J. Klug, S. W. Cheong, M. J. Bedzyk, O. Auciello, D. G. Schlom, R. Ramesh, J. Orenstein, J. L. Musfeldt, V. Gopalan, *Phys. Rev. B* **2009**, 79, 224106.
